# Total or Partial Knee Arthroplasty Trial - TOPKAT: study protocol for a randomised controlled trial

**DOI:** 10.1186/1745-6215-14-292

**Published:** 2013-09-12

**Authors:** David Beard, Andrew Price, Jonathan Cook, Ray Fitzpatrick, Andrew Carr, Marion Campbell, Helen Doll, Helen Campbell, Nigel Arden, Cushla Cooper, Loretta Davies, David Murray

**Affiliations:** 1 Nuffield Department of Orthopaedics, Rheumatology and Musculoskeletal Sciences, Botnar Research Centre, University of Oxford, Headington, Oxford OX3 7LD, UK; 2 Department of Public Health, University of Oxford, Rosemary Rue Building, Old Road Campus, Headington, Oxford OX3 7LF, UK; 3 Health Services Research Unit, University of Aberdeen, 3rd Floor, Health Sciences Building, Foresterhill, Aberdeen, AB25 2ZD, UK; 4 Norwich Medical School, University of East Anglia, Norwich Research Park, Norwich NR4 7TJ, UK

**Keywords:** Medial compartment osteoarthritis, Total knee replacement, Unicompartmental knee replacement, Equipoise, Expertise

## Abstract

**Background:**

In the majority of patients with osteoarthritis of the knee the disease originates in the medial compartment. There are two fundamentally different approaches to knee replacement for patients with unicompartmental disease: some surgeons feel that it is always best to replace both the knee compartments with a total knee replacement (TKR); whereas others feel it is best to replace just the damaged component of the knee using a partial or unicompartment replacement (UKR). Both interventions are established and well-documented procedures. Little evidence exists to prove the clinical and cost-effectiveness of either management option. This provides an explanation for the high variation in treatment of choice by individual surgeons for the same knee pathology.

The aim of the TOPKAT study will be to assess the clinical and cost effectiveness of TKRs compared to UKRs in patients with medial compartment osteoarthritis.

**Methods/Design:**

The design of the study is a single layer multicentre superiority type randomised controlled trial of unilateral knee replacement patients. Blinding will not be possible as the surgical scars for each procedure differ.

We aim to recruit 500 patients from approximately 28 secondary care orthopaedic units from across the UK including district general and teaching hospitals. Participants will be randomised to either UKR or TKR. Randomisation will occur using a web-based randomisation system. The study is pragmatic in terms of implant selection for the knee replacement operation. Participants will be followed up for 5 years. The primary outcome is the Oxford Knee Score, which will be collected via questionnaires at 2 months, 1 year and then annually to 5 years. Secondary outcomes will include cost-effectiveness, patient satisfaction and complications data.

**Trial registration:**

Current Controlled Trials ISRCTN03013488; ClinicalTrials.gov Identifier: NCT01352247

## Background

Osteoarthritis in the knee affects different people in different ways. In the majority of patients with osteoarthritis of the knee the disease originates in the medial compartment [1]. There are varying forms of treatment for this and each aim to relieve pain and discomfort, to reduce stiffness and to minimise further damage to the joint. Such approaches include physiotherapy, drug therapy and surgery [2-5]. One of the surgical options is to replace the diseased joint with a prosthesis (arthroplasty) and there are different approaches in common use. Some surgeons feel that it is always best to replace both the knee compartments with a total knee replacement (TKR). Others feel it is best to replace just the damaged component of the knee with a partial or unicompartmental knee replacement (UKR). There is disagreement among knee surgeons with the majority supporting TKR and the minority UKR. Fewer than 5% of knee replacements worldwide are unicompartmental, although it is thought that up to 30% of patients requiring knee replacements have only unicompartmental disease that would be suitable for a UKR [1,6,7]. This variation in decision-making for patients with medial compartmental arthritis has been well illustrated in a recent linked study [8]. The study showed a high variation in decision-making (TKR or UKR) between four different surgeons (up to 59%). Reassuringly, the consistency of treatment choice (test re-test repeatability) for each individual surgeon remained high. The conclusions were that surgeons, given identical information, do not concur on treatment for patients with the same pathology and that consensus treatment for medial osteoarthritis of the knee remains in question.

There are arguments for both approaches. Both interventions are established and well-documented procedures. Each intervention is considered standard care. There exists little evidence, however, to prove the clinical and cost-effectiveness of either management option and decisions on treatment tend to have a large personal opinion component of the surgeon. The TKR supporters believe that the operation is less complex than UKR and thus, in the short-term TKRs are less susceptible to early problems and failures. They also believe that in the longer term the joint disease will progress to the other, normal, compartments of the knee [9,10], thus a UKR will ultimately fail and require revision to TKR anyway [11,12]. In contrast, the UKR supporters believe the UKR gives faster recovery [7,13], fewer complications [14], superior function[15], is more cost-effective than TKR [16], and it is associated with long-term survival of the joint [6,17].

Current patient management for medial osteoarthritis is based on limited evidence. There have been several individual cohort studies, indirect comparisons and retrospective studies but these have addressed specific aspects and many involve only short-term assessments. Such studies include a comparison between TKR and UKR of the kinematics [18], proprioception [19], ability to kneel [15], ease of revision [20-22] and success or revision after various procedures [23-26], appropriateness for specific pathology [27], accuracy of implantation [28-30] and complications [31,32]. No large, well-powered, multicentre randomised controlled trial has been undertaken to directly compare UKR with TKR. The only other previous attempt at comparing these operations on a large scale was that from one of the comparisons in the Knee Arthroplasty Trial (KAT) [33,34]. However, this comparison failed due to slow patient recruitment. The primary reason for this was that a new minimally invasive technique was introduced for UKR between trial design and recruitment - surgeons chose to learn the new technique rather than recruit for the trial. Joint registry data has shown a trend towards TKR having better survival (as assessed by the rate of revision surgery) [11,12], but other studies are characterised by low level evidence, consensus and peer influence [35-38]. In order to test the validity of these results, further investigation is required. Using an appropriate patient population and long-term assessments, the clinical and cost-effectiveness of both treatment options can be examined.

### Objectives

The primary objective for TOPKAT will be to assess the clinical and cost-effectiveness of TKRs compared to UKRs in patients with medial compartment osteoarthritis of the knee.

Secondary objectives include investigation of complications, patient satisfaction, and the cost implications of the knee replacements for patients and employers as well as healthcare providers.

## Methods/Design

### Study design

The design of the study is a single layer multicentre superiority type randomised controlled trial of unilateral knee replacement patients. The randomised controlled trial design will help reduce and prevent potential bias influencing the evaluation.

Participants will be randomised to either UKR or TKR. The trial has a combined equipoise/expertise approach. It enables surgeons who are not in equipoise to deliver only one of the two operations while also allowing surgeons in equipoise to provide both operations. A surgeon who is in equipoise (‘equipoise surgeon’) and has sufficient experience to perform both TKR and UKR will deliver the allocated operation (UKR or TKR). The same surgeon will perform the operation for both arms of the study.

Not all surgeons are able to exhibit this equipoise. They may hold a preference for one treatment over the other often due to experience/expertise with one type of operation. Interestingly, a surgeon may also believe the patient may benefit from one particular operation even though they may not be able to perform it themselves.

Equipoise is difficult to investigate or establish. Self declaration has been used as the main approach but in order to sufficiently secure this state the following aspects are important:

•The equipoise considered must be patient- or individual-based equipoise rather than an overall or general category equipoise based on operation type. The surgeon must consider their position for each individual patient. Only if they believe that either operation will be suitable for an individual patient can the patient then be recruited.

•No surgeon will ever knowingly perform what they consider a substandard surgical procedure.

**Figure 1:**
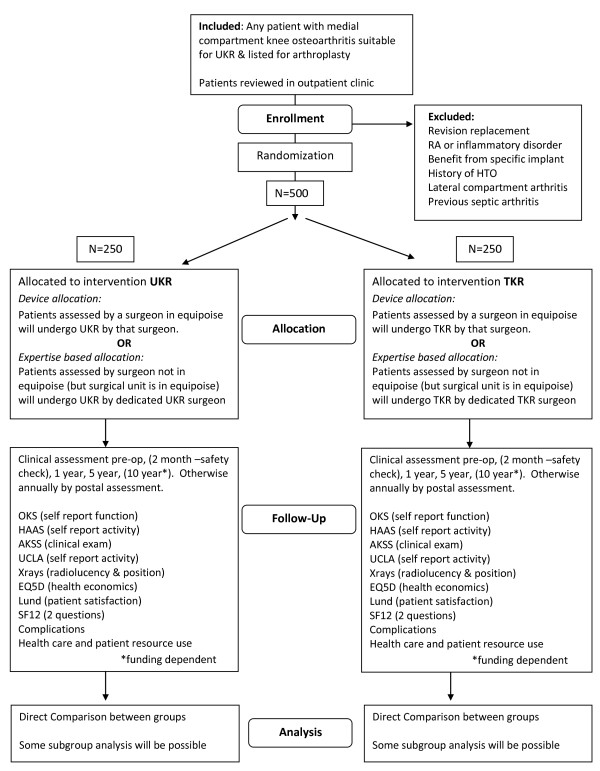
Consort study flow chart.

In order to complete the trial by seeking to maximise surgeon participation, an ‘expertise’-based delivery of the intervention will also occur. For this approach there must be a surgeon with expertise in TKR and a surgeon with expertise in UKR in the same centre who will act together as a ‘delivery unit’. Patients recruited to the study who are under the care of such a surgeon (‘expertise surgeon’) will be randomised to one of the two groups and treated by the appropriate surgeon. This ‘expertise’ approach allows for those UKR surgeons who work alongside TKR surgeons to team up and participate in the study. Subsequent surgery may be carried out by a surgeon different to that at the initial consultation. In such cases the patient is internally referred to the other surgeon’s operating list. A study flowchart is detailed in Figure 1. No restriction is made upon the number of delivery units within a centre. A surgeon can only be in one delivery unit, that is, they are either an ‘equipoise surgeon’ or an ‘expertise surgeon’.

To ensure participating surgeons have appropriate expertise, a simple audit of participating surgeons’ routine practice will be undertaken. UKR surgeons must have had appropriate training, been practising the technique for at least 1 year and have performed the operation at least 10 times in the past year. They must also be aware of their clinical results and these must be acceptable to the study team. Implants used by UKR surgeons in the study must have good clinical results and be a commonly used knee system which does not require patella dislocation. TKR surgeons must satisfy similar criteria. They must have had many years experience with TKR and will use a conventional approach with patella dislocation. ‘Equipoise surgeons’, who deliver both operations, are required to satisfy the criteria for both operations, that is, they will have appropriate training in both operations and have performed a minimum of 10 UKR and 10 TKR procedures.

### Primary outcome measure

The primary outcome measure for TOPKAT is the Oxford Knee Score (OKS). This is a patient-reported outcome questionnaire specifically designed and developed to assess function and pain after TKR surgery; it is a validated and effective measure of change over time [39]. Although patients will be contacted annually, the primary analysis will be at 5 years (and 10 years (subject to additional funding) post randomisation. An early analysis at 1 year is also planned.

### Secondary outcome measures

•American Knee Society Score [40] - measures range of motion and function of the knee

•UCLA Activity Score [41] - measurement of patient activity level in arthroplasty patients with mid/lower level activity

•High Activity Arthroplasty Score (HAAS) [42] - measurement of patient activity accounting for patients with potentially higher levels of activity

•Radiographic features including signs of potential failure, that is, loosening

•EuroQol EQ-5D [43] - evaluation generic measure of health-related quality of life to be used for the economic evaluation

•Lund Score [44] - measurement of patient satisfaction

•Complications

•Length of hospital stay

•Re-operation rate (minor revision, major revision and other related procedure)

•Composite outcome assessment - combination of re-operation frequency and poor outcome in terms of OKS. The anchor based minimally important change (MIC) of the OKS will be used to identify poor outcome (‘lack of success’) for functional outcome [45]

•Adjunct score to the OKS (for younger/active patients)

### Health economics

The health economic evaluation proposed will take the form of a cost-utility analysis. Health outcomes will be assessed at each trial follow-up point using the EuroQol EQ-5D questionnaire and each patient’s resulting utility profile will be used to calculate the number of quality-adjusted life years (QALYs) they experience over the duration of the trial.

To estimate the direct healthcare costs associated with both types of knee replacement, information will be collected for each patient in the trial on the resources consumed during initial surgery (including time in theatre, implants used and hospital inpatient stay), and on any subsequent related healthcare use (for example, relating to complications and/or surgical re-operation including revision). Data on costs patients may incur as a result of their knee condition, including rehabilitation, will be recorded. Information will also be collected from patients on return to paid employment.

### Study setting

Five hundred patients will be recruited from approximately 28 secondary care orthopaedic units from across the UK including district general and teaching hospitals over a period of 3 years.

### Study participants

Participants with osteoarthritis of the medial compartment of the knee will be included in the study. Patients must satisfy general requirements for a medial UKR which are listed below as the inclusion criteria to be recruited to TOPKAT. It should also be noted that if patients meet the inclusion criteria with both their knees, only one knee (designated the study knee) will be operated on according to the randomised allocation assigned as the patient enters into the study. Knee specific measures will be collected (primarily) for the study knee. TOPKAT will not recruit patients with simultaneous bilateral knee replacements. Subsequent knee replacement on the other, non study, knee will be recorded but the subsequent operation will not lead to a further random allocation.

### Inclusion criteria

•Medial compartment osteoarthritis with exposed bone on both femur and tibia

•Functionally intact anterior cruciate ligament (superficial damage or splitting is acceptable)

•Full thickness and good quality lateral cartilage present

•Correctable intra-articular varus deformity (suggestive of adequate medial collateral ligament function)

•Medically fit showing an ASA of 1 or 2

### Exclusion criteria

•Require revision knee replacement surgery

•Have rheumatoid arthritis or other inflammatory disorders

•Are unlikely to be able to perform required clinical assessment tasks

•Have symptomatic foot, hip or spinal pathology

•Previous knee surgery other than diagnostic arthroscopy and medial menisectomy

•Previously had septic arthritis

•Have significant damage to the patella-femoral joint especially on the lateral facet.

### Recruitment and consent

Potential patients will be identified and approached in outpatient and at pre-assessment clinics by the participating surgeon (or their late stage trainee). At this stage patients will be provided with an ‘Invitation letter’ and information sheet which will explain why they have been approached and will provide further details about the study. Patients will indicate if they are willing to be contacted again by the research team, using the TOPKAT Yes/No (‘opt in’) form. Those patients who indicate ‘Yes’ will be contacted by local study staff to arrange a screening visit to assess their eligibility for the study. If the patient is identified during an outpatient appointment the screening visit could coincide with their pre-assessment clinic appointment. The pre-assessment appointments are routinely scheduled for a short time before their scheduled operation date. If patients were identified at their pre-assessment clinic appointment, an extra visit will have to be coordinated for the screening to take place before the patient’s operation date. Contact with the patient must be made at least 48 hours following introduction to the study.

Potential patients may also be identified from local databases. These patients will be sent a letter and a TOPKAT YES/NO form to return documenting if they are willing to be contacted subsequently.

During the screening visit patients will be asked to sign a consent form. This allows their details to be entered into the TOPKAT web-based data collection system. Patient details and all preoperative assessments will be recorded and a study number will be allocated.

Patients will be given sufficient time to accept or decline involvement. They will be free to withdraw from the study at any time without affecting their routine perioperative care.

### Study assessments

Preoperative assessments will include a patient reported questionnaire examining pain and function (OKS), activity level and healthcare resource use. In addition, the American Knee Society Score (AKSS), a clinical assessment of range of motion and function of the knee, will be carried out. Routine preoperative X-rays will also be collected.

Operative details will be recorded and routine postoperative X-rays collected.

Patients will be required to attend a clinic appointment for the AKSS assessment at 2 months, 1 and 5 years post operation. The first two visits will coincide with routine hospital follow-up visits for these knee replacement procedures.

A postal questionnaire (containing the OKS, UCLA, HAAS, EQ-5D, Lund, healthcare and patient resource use) will also be sent out at years 1 to year 5 post randomisation. Additional clinical and postal questionnaire assessments are planned for years 7 and 10 subject to funding. The components of follow-up are shown in Table 1.

**Table 1:** Components of follow-up time points

	**Pre**	**2 months**	**1 year**	**2 years**	**3 years**	**4 years**	**5 years**	**7 years**	**10 years**
OKS (self-report function)	*▲*	*○*	*○*	*○*	*○*	*○*	*○*	*○*	*○*
AKSS (clinical exam)	*▲*	*▲*	*▲*				*▲*		*▲*
UCLA (self-report activity)	*▲*	*○*	*○*	*○*	*○*	*○*	*○*	*○*	*○*
High Activity Arthroplasty Score	*▲*	*○*	*○*	*○*	*○*	*○*	*○*	*○*	*○*
X-rays	*▲*	▲*					*▲*		*▲*
EQ-5D	*▲*	*○*	*○*	*○*	*○*	*○*	*○*	*○*	*○*
Lund (patient satisfaction)		*○*	*○*	*○*	*○*	*○*	*○*	*○*	*○*
Complications		*▲*	*▲*	*○*	*○*	*○*	*▲*	*○*	*▲*
Healthcare and patient resource use	*▲*	*○*	*○*	*○*	*○*	*○*	*○*	*○*	*○*

*▲* = Collected at clinic visit *○* = Postal Questionnaires * = Immediately Post Op.

Due to the variation in waiting times for surgery at participating sites, it is possible that a small number of participants may receive their 1 year follow-up questionnaire (which is sent post randomisation) at a time point much earlier than 1 year post surgery when the clinical assessment is carried out. These patients will have their 1 year assessment too early in their recovery for the results to be valid and there will be great variation in follow-up time. In cases where there is more than 12 weeks between randomisation of a patient to treatment and their operation date, an additional OKS will be administered at the clinical assessment 1 year post surgery.

### Randomisation procedures

Randomisation will occur using a web-based randomisation service at the Centre for Healthcare Randomised Controlled Trials (CHaRT), Health Services Research Unit, University of Aberdeen.

The minimisation algorithm will incorporate gender, age and baseline OKS and ‘delivery unit’. A delivery unit is either an ‘equipoise surgeon’ or a pair of ‘expertise surgeons’ with complementary expertise (that is, one TKR-focused and one UKR-focused).

This factor is included to ensure balance is maintained for individual equipoise surgeons and more generally by centre. Participating surgeons will be discouraged from changing practice during the course of the trial. Within a centre there may be a mixture of delivery unit types. Local recruitment officers at each site will undertake the randomisation. The randomised treatment will be recorded in the patient’s hospital notes and study notes and the surgeon will be notified. If the allocated operation is not provided by the recruiting surgeon (for example, they are an ‘expertise surgeon’ who provide the other operation), an ‘internal referral’ to their delivery unit colleague will be initiated. A standard letter informing the admissions department/care-pathway coordinators will be sent. Local study staff will oversee this referral. Patients’ GPs will also be notified at this time.

### Interventions

TOPKAT will be pragmatic in terms of implant selection for the knee replacement operation. Providing the above conditions are met, surgeons will be entirely free to use an implant of their choice or will use the current implants used at their institution. Implant type used on each patient will be recorded.

#### Total knee replacement

A total knee replacement involves all surfaces of the knee being replaced. The procedure involves excising both diseased and normal femoral condyles, the tibial plateau and often the patella. This is done through a large skin incision which provides easy access to the knee joint. Each component will be replaced with an artificial implant, which may be cemented in position.

#### Partial knee replacement

A partial knee replacement or UKR involves only the diseased area of the joint being replaced. The healthy compartment of the knee is retained and artificial implants are inserted in place of the diseased area. This is done via a minimally invasive surgical procedure.

### Safety

The TOPKAT trial involves routine knee replacement surgery for medial compartmental osteoarthritis. There are no additional risks to patients. They will undergo knee replacement as per standard management regime. The benefits will be to future patients although involvement in the trial with specific outcome measurement may be perceived as a benefit by some patients. Patients will be informed of the standard risks associated with anaesthetic and knee replacement operations.

Possible (expected) complications and consequences are:

•All substantive surgical procedures (including knee replacement) whether primary or revision procedures carry a risk of anaesthesia-related problems, death, morbidity including wound infection, bleeding intra- and postoperatively, thrombo-embolic complications and complications secondary to existing co-morbidity, for example, ischaemic heart disease.

•Specific complications following knee replacement procedures include loosening of components - tibia/femur/both, dislocation of knee/bearing, superficial and deep infection, unexplained knee pain, knee stiffness, haematoma, mechanical failure of replacement, periprosthetic fracture. These complications may result in the need for further surgery such as revision operations, arthroscopy, washout, manipulation under anaesthetic, debridement (open), aspiration, above knee amputation, patella resurfacing.

For the purpose of TOPKAT, a serious adverse event (SAE) is defined as any adverse event during the course of the study resulting from the administration of any of the research procedures required by the protocol that:

•Results in death,

•Is life-threatening†,

•Requires inpatient hospitalisation or prolongation of existing hospitalisation,

•Results in persistent or significant disability/incapacity, or

•Other important medical events*

† The term ‘life-threatening’ in the definition of ‘serious’ refers to an event in which the participant was at risk of death at the time of the event; it does not refer to an event which hypothetically might have caused death if it were more severe.

*Other events that may not result in death, are not life-threatening, or do not require hospitalisation, may be considered a SAE when, based upon appropriate medical judgement, the event may jeopardise the participant and may require medical or surgical intervention to prevent one of the outcomes listed above.

All SAEs will be notified to the appropriate authorities (Research Ethics Committee (REC) and Sponsor) within the timelines specified.

When the web-based SAE form is completed detailing any possible related and unexpected SAEs, the Chief Investigator (CI) or deputy will be notified automatically. If, in the opinion of the local surgeon and the CI, the event is confirmed as being related and unexpected (that is, not listed as a possible expected occurrence), the CI will submit a report to the main REC and the study sponsors within 15 days of the CI becoming aware of it.

The reporting procedures for all study-related adverse events are detailed in accordance with the guidance from the National Research Ethics Service (NRES).

### Reporting of postsurgical complications

The annual postal self-report questionnaires will ask patients if they have been admitted to hospital at any point over the last 12 months. Any readmissions will be followed up by the trial coordinator in Oxford who will contact the recruitment officers at the patient’s hospital and ask them to collect further information about the readmission event. Details of any readmissions that are study-related (that is, result from administration of any of the procedures required by the trial protocol) and are expected (that is, listed as a possible expected occurrence) will be collected.

At the routine follow-up clinical visits, patients will also be asked if they have experienced any complications related to their study knee since their last scheduled TOPKAT visit, which resulted in them visiting a healthcare practitioner. This information will be recorded.

### Statistics and analysis

#### Sample size

The sample size for the trial (250 in each arm, 500 overall) has been based on a number of considerations, drawing on what previous research has suggested is both plausible and the likely size of difference that is clinically significant.

#### Primary outcome - OKS score

Table 2 shows the number of subjects required in each randomised group to give either 80% or 90% power to detect differences in the OKS of 2.0, 3.0 and 4.0, at either the two-sided 1% or 5% significance level and with SD of 8.0, 9.0 or 10.0.

**Table 2:** Sample size scenarios

**Number in each group**		**Mean difference in OKS**					
		**2.0**		**3.0**		**4.0**	
**Power**	**SD**	**1% sig level**	**5% sig level**	**1% sig level**	**5% sig level**	**1% sig level**	**5% sig level**
90	8.0	480	340	215	150	120	85
	9.0	600	430	270	190	150	110
	10.0	740	520	330	235	190	130
80	8.0	375	250	170	110	100	60
	9.0	470	320	210	140	120	80
	10.0	590	390	260	175	150	100

The minimal clinically significant difference of the OKS is judged to be 2.0, and the likely SD of the OKS is 8.0 [46]. A sample size of 500 patients (250 in each group) would provide 80% power to detect a difference of 2.0 at 5% (two-sided) significance level. Since it is possible that the SD of the OKS could be >8.0 [13], this size of sample would allow for the detection of a difference of 3.0 in OKS with a SD of 10.0 at 5% (two-sided) significance level and 90% power and also a difference of 3.0 at 1% (two-sided) significance level with 80% power.

Indeed, almost all of the above scenarios are detectable if the difference in OKS is 3.0 rather than 2.0. This difference of 3.0 in the OKS is equivalent to a typical category change in the American Knee Society Score [40]. Furthermore, a difference in the OKS of 4.0 would, with 250 patients per group allow for some subgroup analyses. As previous research (the Bristol RCT) suggests that the difference between the groups is indeed likely to be >2.0 [6], a sample size of 250 in each arm would allow for some non-response yet still detect differences. As the statistical analysis will adjust for the baseline value and account for the surgical delivery unit this will likely increase precision. Offsetting this will be any missing data which would have the reverse impact.

#### Re-operation (including revision) of the device

UKR may be associated with higher re-operation rates, including revision. The re-operation rate after TKR is anticipated to be approximately 5% at 5 years [47]. A sample size of 250 patients per group would give 80% power at *P* <0.05 to detect an increase to 12% (compared to just under 5%), and 90% power at *P* <0.05 to detect an increase to 14%. Analysis based on the time to re-operation using survival analysis will likely be more than sufficient.

### Statistical analysis

Principle analyses will be based on an ‘intention to treat’ basis where participants will be analysed according to the allocated group using all available participant data. Statistical significance will be judged at the two-sided 5% level with corresponding 95% confidence interval presented. A short summary of the proposed analyses is given below. Further details of the planned statistical analyses are contained in the Statistical Analysis Plan, which will be finalised, prior to the unblinding of data.

Three sets of analyses are planned, based on the anticipated follow-up period. By 2 years, all patients are anticipated to have received surgery. Analyses are planned at 1 year post operation (3 years into the trial), at 5 years randomisation (7 years into the trial) and 10 years post randomisation (12 years into the trial).

Under the principal analysis of the primary outcome, the OKS score will be compared at each assessment point alone (multiple linear regression analysis adjusted for minimisation factors). The impact of adjusting for delivery unit will be explored using multilevel modelling. A stratified analysis (and associated interaction effect) will be performed to allow for a difference between expertise versus equipoise delivery of the treatments and the potential impact upon the comparison. Secondary analyses will explore the potential impact of missing data and alternative analytic approaches. In addition to the analyses planned once 5 and 10 year follow-up has matured, a complementary analysis will also compare the OKS over all assessments (the follow-up period) using multilevel modelling to allow for repeated measurements for participants. One secondary analysis, using an external comparative cohort, will also evaluate whether trial patients are representative, in terms of age, patient demographics and operative findings, of patients undergoing UKR and TKR in the general population.

Secondary outcomes will be analysed in a similar manner adjusting for minimisation factors where appropriate within a generalised linear models framework. Confidential interim analysis will be performed as requested by the Data Monitoring Committee.

For the health economic evaluation within-trial cost-effectiveness will be assessed at 5 years (and at 10 years - subject to additional funding). Costs and QALYs in each trial arm will be expressed using means and standard deviations. Incremental analysis will be performed. If appropriate, results will be expressed as an incremental cost per QALY gained, with uncertainty around this ratio determined through the use of non-parametric bootstrapping and cost-effectiveness acceptability curves [48,49]. Longer-term extrapolation of results will also be conducted and will use trial data, for example surgical re-operation rates will be projected using a simple parametric model and will be assigned appropriate event costs and utility scores.

### Ethics

The study obtained approval from the National Research Ethics Service, Oxfordshire REC C in September 2009 (09/H0606/88).

### Trial status

Recruitment to the TOPKAT trial commenced in January 2010 and is ongoing at the time of manuscript submission. To date, 478 patients have been randomised (May, 2013). Recruitment was originally scheduled to end in December 2011. Early indications were that recruitment targets would not be met due to early delays with R&D approval process and time taken to incorporate the study procedures into the routine practice of participating sites. An extension to recruitment was discussed and recommended by the TMG and TSC which was agreed upon by the funders. The recruitment phase was extended to September, 2013.

## Abbreviations

AKSS: American knee society score; HAAS: High activity arthroplasty score; KAT: Knee arthroplasty trial; OKS: Oxford Knee Score; QALYs: Quality adjusted life years; TKR: Total knee replacement; UCLA: University of California Los Angeles; UKR: Unicompartment replacement.

## Competing interests

Professor David Murray receives royalties from Biomet (the company who make the Oxford Unicompartmental Knee Replacement system). Professor Andrew Price receives consultancy fees from Biomet. However, TOPKAT does not actively promote the use of one unicompartmental system over another. All unicompartmental systems may be used as part of the study.

Jonathan Cook holds a Medical Research Council Methodology Fellowship (G1002292). The Health Services Research Unit is core funded by the Chief Scientist Office of the Scottish Government Health Directorates. Views expressed are those of the authors and do not necessarily reflect the view of funders.

## Authors’ contributions

DB as Chief Investigator, AP, JC, RF, AC, MC, HD, HC, NA and DM were co-applicants on the grant application to the NIHR HTA, and were involved in the design of the study and its implementation, as was CC. LD is the study co-ordinator. DB, JC, DM, HC and LD were responsible for writing this manuscript. All authors read and approved the final manuscript.

## Authors’ information

David Beard is the Chief Investigator of the study, Professor of Musculoskeletal Sciences and Director of OSIRIS (Orthopaedic Surgical and Interventional Trials Unit) within the Nuffield Department of Orthopaedics, Rheumatology & Musculoskeletal Sciences at the University of Oxford.

Andrew Price is co-first author, Professor of Orthopaedic Surgery within the Nuffield Department of Orthopaedics, Rheumatology & Musculoskeletal Sciences at the University of Oxford.
